# The Microbe Directory: An annotated, searchable inventory of microbes’ characteristics

**DOI:** 10.12688/gatesopenres.12772.1

**Published:** 2018-01-05

**Authors:** Heba Shaaban, David A. Westfall, Rawhi Mohammad, David Danko, Daniela Bezdan, Ebrahim Afshinnekoo, Nicola Segata, Christopher E. Mason

**Affiliations:** 1 Department of Physiology and Biophysics, Weill Cornell Medicine, New York, NY, 10065, USA; 2 The HRH Prince Alwaleed Bin Talal Bin Abdulaziz Alsaud Institute for Computational Biomedicine, Weill Cornell Medicine, New York, NY, 10065, USA; 3 CUNY Hunter College, New York, NY, 10065, USA; 4 School of Medicine, Weill Cornell Medicine, New York, NY, 10065, USA; 5 CUNY College of Staten Island, Staten Island, NY, 10314, USA; 6 School of Medicine, New York Medical College, Valhalla, NY, 10595, USA; 7 Centre for Integrative Biology, University of Trento, Trento, 38122, Italy; 8 The Feil Family Brain and Mind Research Institute, New York, NY, 10065, USA

**Keywords:** Microbe, Metagenomics, Microbiome, Next-Generation Sequencing, Metadata, Database

## Abstract

The Microbe Directory is a collective research effort to profile and annotate more than 7,500 unique microbial species from the MetaPhlAn2 database that includes bacteria, archaea, viruses, fungi, and protozoa. By collecting and summarizing data on various microbes’ characteristics, the project comprises a database that can be used downstream of large-scale metagenomic taxonomic analyses, allowing one to interpret and explore their taxonomic classifications to have a deeper understanding of the microbial ecosystem they are studying. Such characteristics include, but are not limited to: optimal pH, optimal temperature, Gram stain, biofilm-formation, spore-formation, antimicrobial resistance, and COGEM class risk rating. The database has been manually curated by trained student-researchers from Weill Cornell Medicine and CUNY—Hunter College, and its analysis remains an ongoing effort with open-source capabilities so others can contribute. Available in SQL, JSON, and CSV (i.e. Excel) formats, the Microbe Directory can be queried for the aforementioned parameters by a microorganism’s taxonomy. In addition to the raw database, The Microbe Directory has an online counterpart ( https://microbe.directory/) that provides a user-friendly interface for storage, retrieval, and analysis into which other microbial database projects could be incorporated. The Microbe Directory was primarily designed to serve as a resource for researchers conducting metagenomic analyses, but its online web interface should also prove useful to any individual who wishes to learn more about any particular microbe.

## Introduction

With the advent of next-generation sequencing technologies, there has been a surge of metagenomic and microbiome studies in the last decade, ranging from studying the human microbiome ^1^ to the environment (water and soil) ^2– 5^, and city surfaces ^6, 7^. All these studies depend heavily on bioinformatics analyses that translate the sequences they uncover to taxonomic profiles found in their samples. However, an immediate challenge from taxonomoic outputs is the interpretation of the data. Learning more about a microorganism’s properties, such as optimal pH and temperatures, presence in the human microbiome, ability to form spores or biofilms, and antimicrobial sensitivity, amongst many others, are key to understanding the biochemical and ecological dynamics of the microbiomes that can be found. Despite the presence of several databases that include some of this information, such as MicrobeWiki, PATRIC, ARDB, and IMG-JGI, these databases are either incomplete or focus on a specific characteristic (e.g. antimicrobial resistance). The Microbe Directory seeks to fill this gap with an online tool that aggregates these data and expands their annotations, which thus provides a useful tool for exploration of functional, medical, or biological traits found in any microbial community.

## Methods

### MetaPhlAn2 list of species

The list of distinct species that was subject to curation was generated from the MetaPhlAn2 database, a computational tool for profiling the composition of microbial communities from sequencing data. MetaPhlAn2 works by relying on unique clade-specific marker genes identified from more than 16,000 reference genomes from NCBI and RefSeq ^8^. It provides a 7-level (kingdom to strain) consistent taxonomic characterization of known domains of life and currently has identified >7,500 unique species in its database. This database was specifically chosen for the Microbe Directory due to its prevalent usage in microbiome and metagenomic studies ^9^, allowing researchers to directly integrate the Microbe Directory into their research to learn more from the MetaPhlAn output ^10^. Furthermore, there is a built-in capability for researchers to contribute and expand the Microbe Directory beyond the species currently curated in the database (see *Using the Microbe Directory*).

### Selection and training of researchers

The Microbe Directory database was curated by a team of trained undergraduate, graduate, and medical students from City University of New York (CUNY) Hunter College, Macaulay Honors College, and Weill Cornell Medicine (see full list of students in *Acknowledgements*). The student-researchers were selected from a pool of applicants and underwent a three-hour training session that a) explained the objective of the research project and the desired outcome, b) provided a detailed and thorough explanation of each of the parameters that were the subject of research, and c) provided clear instructions on how to curate the internet for the parameters for each species. They were also given a tutorial on how to conduct the research for a sample of 10 species. They were given a list of annotation-based websites to assist their research, but they were not limited to using only those sites. (see *Annotation Tutorial and Guidelines* in Supplementary File 1).

After every entry, students inserted citation links to the sources they utilized for the information they inputted. Each student-researcher independently worked 4–5 hours per week to curate parameters for 10 species per week, for a total of 20 weeks. To ensure that students were not making errors during curation, the first three weeks of the project were heavily monitored and entries were manually checked for inaccuracies by the project leads. After the first 3-week trial, only two randomly selected species were checked manually from every submitted entry of 10 species per week, per student. Considerable error rates (3 or more incorrect annotations out of 10 being the threshold) consequently meant the student had to resubmit the entire set of 10 species the following week. While there is always the potential for human error in manually curated databases, the Microbe Directory has a feature where anyone can make an account and submit edits and changes to the information hosted in the database. Thus, there is potential for the Microbe Directory to continue to grow and expand, but also ensure minimal errors in its database.

### Building the microbe directory

**Figure 1.**
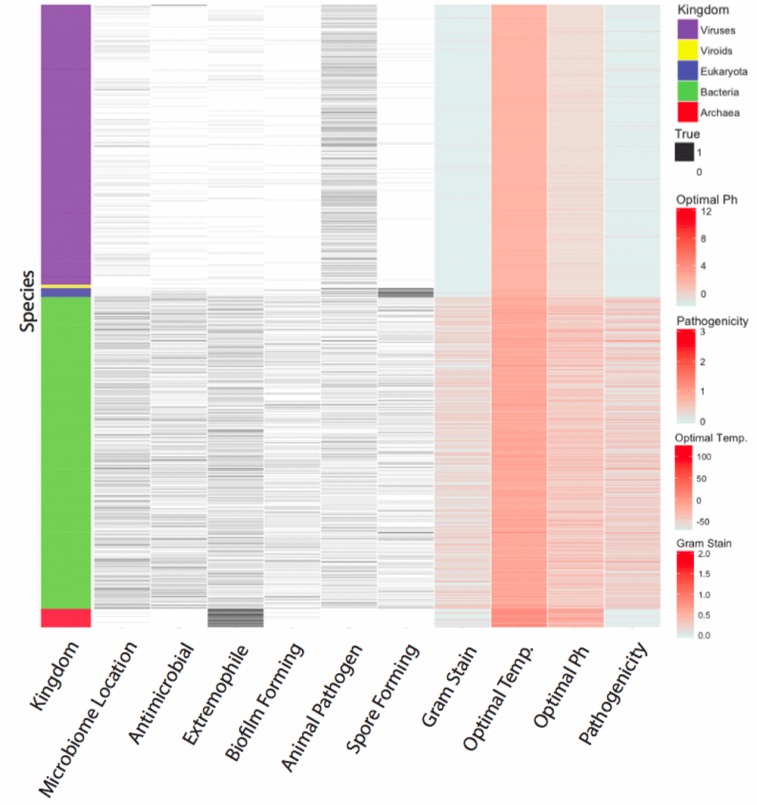
Microbe Directory heatmap. Annotation types (x-axis) are represented across the online database and the numbers of each category (y-axis, left side) are shown, including Viroids (purple), Viruses (yellow), Eukaryotes (blue), Prokayotes (green), and Fungi (red). The scale for each of the types of metadata (right) are also shown for binary classifications (black, white) and quantitative traits (red scales). Heatmap was constructed using R (version 3) and Illustrator.

Table 1 defines the various microbial characteristics and categories of information that were curated to build the Microbe Directory. The parameters chosen were strictly objective features of microbes that are important to help interpret and understand the findings and context of whatever microbiome a researcher is studying. There is built-in potential to expand the Microbe Directory and for researchers to contribute more characteristics of these microbes, including native location, industrial applications, and associated symptoms/diseases; these features were considered to be included in the Microbe Directory but due to their subjective nature were omitted out to maintain proper quality control outlined above. Several databases were used to collect this information, including COGEM, MicrobeWiki, BacMap, ATCC, PATRIC, ARDB, GOLD, HOMD, and BEI Resources (see *Annotation Tutorial and Guidelines and Links* in Supplementary File 1). These peer-reviewed resources and databases have been well-established in the literature as reliable sources of information for researchers. Now, this information can be housed in one place, allowing for more efficient and comprehensive interpretation of microbiome analysis. Figure 1 is a heatmap summarizing the current information hosted in the Microbe Directory’s database across all species and parameters.

**Table 1.** The Microbe Directory inventory parameters and descriptions.

Parameter	Definition and notes
**Optimal pH**	The optimal pH at which this species grows. If the species was not widely studied, the American Type Culture Collection (ATCC) was used to determine the optimal pH for storage. If two far ranges of pH were determined, the average was taken.
**Optimal** **temperature**	The optimal temperature at which this species grows. If the species was not widely studied, the ATCC was used to determine the optimal temperature for storage. If two far ranges of temperatures were determined, the average was taken.
**COGEM** **pathogenicity** **rating**	COGEM released a comprehensive database of pathogenicity assessment of around 2575 bacterial species in 2011 ^10^. The database ranks the pathogenicity of species on a scale of 1 to 4 - 1 being not belonging to a recognized group of disease-invoking agents in humans or animals and having an extended history of safe usage and 4 being a species that can cause a very serious human disease, for which no prophylaxis is known.
**Antimicrobial** **susceptibility**	Are there any known antibiotics that this species is sensitive to? **No = 0, Yes = 1**
**Spore-formation**	Is the species spore-forming? **No = 0, Yes = 1**
**Biofilm-formation**	Is the species biofilm-forming? **No = 0, Yes = 1**
**Extremophile**	Extremophiles are organisms that live in extreme environments, as opposed to organisms that live in moderate (mesophilic) environments. This category includes acidophiles, thermophiles, osmophiles, halophiles, oligotrophs, and others. **Mesophiles = 0, Extremophile = 1**
**Gram-stain**	**Negative = 0, Positive = 1, Indeterminate = 2**
**Found in human** **microbiome**	Microbes that live anywhere in the human body and are not pathogenic to humans (i.e. capable of causing human disease) **No=0, Yes=1**
**Plant pathogen**	Does the species causes disease in plants? **No = 0, Yes = 1**
**Animal pathogen**	Does the species causes disease in animals? **No = 0, Yes =1**

***Pre-search*.** Before assignments were given to the student-researchers, the databases listed above were pre-searched in order to collect as much information as possible about the microbes. This was done using each website's search page. The species name was used as the search query, and the search results html page was parsed using regular expressions. The first search result that contained the microbe's binomial name and contained a link to the website's entry for that microbe was used as the pre-search's result. Such links for each microbe were compiled and given to each student with his or her weekly assignments. The student-researchers were only given the link to the entry, and they then had to manually find the relevant information (e.g. "optimal pH"). Such a system allowed the students to manually confirm that the pre-search identified the correct entry for the microbe and not just a microbe with a similar name. We also supplemented the manual curation by parsing MicrobeWiki for common keywords that could indicate particular features. We found that we could extract useful data for pathogenicity, biofilm-formation, microbe shape, halophilicity, spore formation, and metabolism. We were able to extract some subset of these features for 331 of the microbes that had been manually curated.

***Text validation and normalization*.** Student-researchers filled out the columns for a given microbe using an Excel spreadsheet. Each entry was filled out as free-form text, so it was necessary to later normalize and validate the text. Valid column types included positive real numbers (e.g. optimal pH), ranges of positive real numbers (e.g. range of optimal pH values), series of ranges (e.g. multiple optimal pH ranges), binary values (e.g. spore forming or non-forming), ternary values (e.g. Gram-positive, Gram-negative, Gram-indeterminate), and quaternary values (e.g. COGEM Classes 1-4). Regular expressions (RegEx) were used to ensure that a given column entry conformed to the correct type (i.e. validation); validated columns were then transformed to a common form (i.e. normalization). The common form for each entry is the form used in the database.

### Using the Microbe Directory

The Microbe Directory can be accessed online at https://microbe.directory. This interface provides individual users a way to browse and search the directory’s contents in an interactive format. Such a representation should prove useful for researchers who need information for a particular microbe. While viewing the page for a given microbe, registered users can also submit edits to that microbe’s data. Individuals can register to contribute to the Microbe Directory by signing up here. The edits are then put in a queue to be later reviewed by The Microbe Directory team (HS, DAW, RS).

In addition to the interactive web interface, the main website provides links to the project’s GitHub and BitBucket repositories. From the GitHub repository, users can download the SQLite database used to power the website. Users will also find JSON and CSV (i.e. Excel) representations of the database, which are auto-generated from the SQLite database using Python scripts. Since the Microbe Directory is meant to grow and expand over time, researchers wanting to make more substantial contributions can suggest changes to the database through our GitHub page. The requested changes will be merged as appropriate and could be incorporated into future releases. Moreover, there is a tutorial on the GitHub repository that shows users how they can use the JSON version of the database given a MetaPhlAn2 output file. Finally, the website used to power the web interface can also be accessed and modified through a separate BitBucket repository, which can also be accessed through the main website.

The Microbe Directory was designed to help researchers in the microbiome and metagenomics fields to learn more about the organisms they are identifying through their bioinformatics analyses. While this is only version 1.0 of the Microbe Directory, it is readily able to incorporate any contributions to the database to expand the microbial features included in our inventory. For more information on how to contribute to the project visit https://microbe.directory/.

## Data availability

The web interface for the Microbe Directory can be found at https://microbe.directory/

The database and other files can also be found on the GitHub repository here: https://github.com/microbe-directory/microbe-directory and the BitBucket repository here: https://bitbucket.org/account/signin/?next=/microbedb/microbedb. *Note:* BitBucket requires a login, but account generation is free and there are no restrictions for signing up.

Archived code as at time of publication:

Github: https://doi.org/10.5281/zenodo.1069858 ^12^
Bitbucket: https://doi.org/10.5281/zenodo.1069860 ^13^

License: MIT

## Supplementary material

**Supplementary File 1: Microbial Annotations - Tutorial and guidelines for student researchers, and useful links and tips.**

**Supplementary File 2: Annotations_Automator.py – Python script used for automated research.**
