# Supplementary material for: The Microbe Directory: An annotated, searchable inventory of microbes’ characteristics
Source: Gates Open Res. 2018 Jan 5;2:3. [Version 1] doi: 10.12688/gatesopenres.12772.1 (PMC5883067; doi:10.12688/gatesopenres.12772.1)
Supplement: Supplementary file 1 [file gatesopenres-2-13832-s0000.tgz › 1a7798a3-a13e-402b-9f1d-52f341001aa9.pdf]

Supplementary File 1.

Microbial Annotations- Tutorial and Guidelines for Student Researchers

PARAMETERS

Optimal pH

Use ATCC, as if ordering a sample of the species. See ‘Useful Tips’

Optimal Temperature

Check lab studies that culture that type of species or use ATCC.

COGEM Pathogenicity Rating

What is its COGEM rating? If only the genus rating is given, denote the number and put ‘genus’ in parenthesis. **Note:** You are looking at the *overall* rating. It is the last, bolded number in the row.

0	1 (genus)
0	1 (genus)
0	1 (genus)
0	1 (genus)
0	1 (genus)
0	1 (genus)
0	1 (genus)
0	1 (genus)
0	1 (genus)

RICKETTSIA HELVETICA	-	2	-	2	2	2	2	2	3	3	**3**
RICKETTSIA HONEI	-	-	-	2	2	2	2	2	3	3	**3**
RICKETTSIA JAPONICA	-	3	-	3	2	2	2	2	3	3	**3**
RICKETTSIA MASSILIAE	-	2	-	1	2	2	2	2	3	3	**3**

Antimicrobial susceptibility

Are there any known antibiotics that this species is susceptible to? No=0, Yes=1

Spore-formation

No=0, Yes=1 (usually same for the whole genus, but not always)

Biofilm-formation

No=0, Yes=1

Extremophile

Extremophiles are organisms that live in extreme environments, as opposed to organisms that live in moderate (mesophilic) environments. This category includes acidophiles, thermophiles, osmophiles, halophiles, oligotrophs, and many others. If the microbe is an extremophile, type ‘1’ in this column; if not, type ‘0’.

Gram stain

Negative= 0, Positive=1, Indeterminate =2 (usually same for the whole genus, but not always)

Found in Human Microbiome

This column is for microbes that live anywhere in the human body and ***are not*** pathogenic to humans (i.e. capable of causing human disease). No=0, Yes=1

Plant pathogen

Is it an organism that causes disease in plants? No=0, Yes=1

Animal pathogen

Is it an organism that causes disease in animals? No=0, Yes=1

LINKS

1. http://bacmap.wishartlab.com/
Useful for: location, gram, metabolism, optimal temperature, habitat, biotic relationship, sporulation, pathogenicity

2. https://microbewiki.kenyon.edu/index.php/MicrobeWiki
Useful for: metabolism, pathogenicity, optimal temperature, location, habitat, biofilm, sporulation, pathogenicity, other uses, ecology

3. https://www.atcc.org/
Useful for: strain #, applications, temperature

4. http://www.mgc.ac.cn/VFs/search_VFs.htm
Useful for: virulence factors, pathogenicity

5. https://www.patricbrc.org/portal/portal/patric/Home
Useful for: virulence factors, pathogenicity, antibiotic resistance, gram, temperature, oxygen requirement

6. http://hmpdacc.org/
Useful for: human microbiome, pathogenicity, temperature

7. http://ardb.cbcb.umd.edu/
Useful for: antibiotic resistance

8. http://jb.asm.org/
Useful for: location, other uses, pathogenicity, metabolism. oxygen requirement, gram, etc.

9. http://gutpathogens.biomedcentral.com/
Useful for: pathogenicity, location, etc.

10. http://www.phidias.us/
Useful for: pathogenicity

11. https://gold.jgi.doe.gov/
Useful for: biotic relationship, disease, optimum temperature, sporulation, pH, oxygen requirement, gram

12. http://www.homd.org/
Useful for: human microbiome

13. https://www.beiresources.org/
Useful for: pathogenicity, etc.

14. http://www.microbiologyresearch.org/
Useful for: pathogenicity, literature, etc.

15. NCBI database
Useful for: location, sporulation, temperature, literature, etc.

16. COGEM PDF
Useful for: pathogenic rating

USEFUL TIPS

1) It is easier to go by columns and then fill in the remaining info row to row, that way you do not have to keep switching back and forth between databases. We suggest downloading the Human Microbiome Database and using the COGEM PDF to start with and filling that information in. Then you could move on to temperature, sporulation, gram stain, pathogenicity. The average search time **per species is about 30 minutes.**

2) If a bacterium is an extremophile, it should be well known. At the very least, its genus will be well known. It is likely you will be able to find all the information you need on a website that deals with well-known bacteria, like Microbewiki.

3) If you find two different websites are giving you contradicting information, it is likely because strains within the species have varying characteristics. (ex. Some strains may grow at different pH's than others). **That is why it is important that you cite every entry in every cell and note discrepancies!**

4) For more well-known bacteria, the most reliable information comes from scientific papers, so download different literature from the NCBI database or another source.

5) If you cannot find growth conditions (optimal temperature and pH) try "ordering" the bacteria online and seeing what growth medium they will package it in (use www.atcc.org)

6) Utilize the NCBI database as much as you can.

7) Keep opening tabs and do not close them because you will have to copy and paste all the websites you used into the citations columns.

8) It is common for articles to focus on either a specific strain or a species as a whole. It is okay to put N/A in a column if you have searched for more than 10 minutes per cell and have not found any information online or in journals.
