# Supplementary material for: The Microbe Directory: An annotated, searchable inventory of microbes’ characteristics
Source: Gates Open Res. 2018 Jan 5;2:3. [Version 1] doi: 10.12688/gatesopenres.12772.1 (PMC5883067; doi:10.12688/gatesopenres.12772.1)
Supplement: Peer review history

# Peer review: Referee response for version 1

**DOI:** 10.21956/gatesopenres.13832.r26308

**Published:** 2018-03-22

**Authors:** Elisabeth M. Bik

**Affiliations:** 1 uBiome, San Francisco, CA, USA

Shaaban *et al.* describe The Microbe Directory, a database with more than 7,500 microbial species. This is a great initiative, in which a group of academic researchers, helped by a team of (under)graduate students have annotated bacteria, archaea, viruses and eukaryotic microbes taken from the MetaPhlAn2 database, with respect to pathogenicity, growth characteristics, and presence in the human microbiome. The paper is well written, and the initiative is very welcome. Although the initial list of fields is small, this database has the potential to grow, and there is an option for registered users to add missing data. 

 Comments on the manuscript:

  

1. A typo in the Introduction: "taxonomoic"
2. In the Methods, section “MetaPhlAn2 list of species”, it reads "7-level (kingdom to strain)". However, based on the MetaPhlAn2 documentation, this should read "kingdom to species" (1, Kingdom; 2, phylum; 3, class; 4, order; 5, family; 6, genus; 7, species).
3. Figure 1.
  1. There is a discrepancy to the color codes described in the legend text and those in the key on the top right of the figure. Viruses, viroids have opposite colors, and the "prokaryotes"  and fungi are not mentioned in the key. 
  2. "Prokaryotes" is not a good term, as it defines the 2 groups by something they do not have, and suggests a common ancestor between archaea and bacteria. See e.g. Norm Pace's essay ^1^.
  3. What is meant by "Microbiome Location"? It has a binary value, suggesting that maybe "Found in Human Microbiome" (as used in Table 1) might be a better description. The “Location” suggests that this field stores which anatomical site this species has been found, which would also be a nice field to have, but not what is meant here.
  4. Similar question for "Antimicrobial" - does a yes mean that it is sensitive or resistant? Or that antimicrobial properties are known? Or that it makes an antimicrobial? Table 1 provides the answer to the question, but it might be worth addressing this here as well.
  5. “Optimal Ph" should be “Optimal pH".
  6. The order of the last 4 categories is different between the labels under the heatmap and the key on the right.
4. A possible and useful addition to the paper would be to describe some potential fields that could be added to the database. In its current form, the useability of the database is very limited, and it would probably be faster to just look up the information in e.g. Wikipedia. But the strength of this database is that it can grow, both in number of entries, as well as number of fields. Some suggestions would be: a link to the draft genome of the organism, number of chromosomes, linear/circular chromosome, RNA/DNA virus.
5. The paper could also address that some information that appears to be simple at first glance, such as pathogenicity, might not be simple at all. For example, Escherichia coli and Clostridium difficile can both be a peaceful member of the human gut microbiota, or a human pathogen, depending on the presence of toxin genes. Herpes virus infections are so common in humans, and usually latent, that one could argue that it might be considered part of the human microbiome. The ability to form biofilms might be also more complicated than just a simple yes/no. The paper would be stronger if it acknowledges the difficulties of capturing these subtleties into simple binary answers.

 

 Comments on the database:

1. Of course, this is just a first version, and the database will hopefully grow quickly, but the current data felt very sparse. For example, and as also pointed out by other reviewers, pH/temperature information was missing for well-studied microbes such as Salmonella enterica, Agrobacterium tumefaciens, Candida albicans, Schizosaccharomyces pombe, or Yersinia pestis. For others, there was an entry present in the database but all fields were empty (e.g. Rhinovirus A, Bacillus cereus thuringiensis). 
2. There are important classification errors such as:
  1. Yersinia pestis, which causes disease in rodents, is not listed as an animal pathogen.
  2. Magnaporthe oryzae, causative agent of one of the most destructive diseases in rice, was not listed as a plant pathogen. 
  3. Candida albicans was listed as not susceptible for antimicrobials, with a reference from 1999.
  4. Influenza B virus is classified as biofilm forming based on a paper that shows that Influenza A virus can disperse Streptococcus pneumonia biofilms.
  5. Agrobacterium tumefaciens, a well-studied plant pathogen, is listed as an animal pathogen, although it does not infect animals in nature, only under laboratory conditions. 
  6. Human herpesvirus 4 (Epstein-Barr virus, but that search did not bring up any results), is not listed as a human pathogen.
3. The “Found in the human microbiome” category is defined as “Microbes that live anywhere in the human body and are not pathogenic to humans (i.e. capable of causing human disease) No = 0, Yes = 1”. However, both Escherichia coli and Clostridium difficile, which can be both a pathogen as well as a symbiotic member of the microbiome, are classified as a “yes” . So maybe the definition of this should be refined and the part about not being pathogenic to humans should be taken out?
4. Links: Fields with data are marked with an “i”, which will lead to the source. Some of the i’s are yellow, while others are black/white. The yellow i’s lead to an URL that is not a hyperlink, while the white i’s are hyperlinks. As one of the other reviewers pointed out, it would be nice if all links would be hyperlinks. Also, in these links, it was very noticeable that the database was compiled of contributions by many different users, who all had their own specific way of adding links. In some cases, the yellow I’s give a citation but they are not very helpful. Examples:
  1. Akkermansia muciniphila: all information links give “Everard, Amandine, et al”, without doi, year, or working link. 
  2. Cryptococcus neoformans: all I’s lead to “Todd W., Larimer, Frank W., Lippmeier, J. Casey, Lucas, Susan, Medina” - no link, year, doi. Which paper is that? I could probably find it, but that defeats the purpose of having a reference database.
  3. Magnaporthe oryzaeAbx susceptibility is listed as an unhelpful “Choi J et al”. A Pubmed search for that author returns 19067 papers .
  4. Escherichia coli: the field for the optimal pH leads to a biotech company that sells culture media, but the link is broken. 
  5. All i’s for Candida albicans refer to Staab, J. F. (1999) ^2^, which albeit a paper about Candida, is an older paper about a specific protein, not a general review paper.
  6. The “Pathogenicity” appears to always use the COGEM 2011 list as a source, but it is annotated in many different ways. It seems that each of the (under)graduate students used a different description for this field. Sometimes it is a working link to the COGEM document, but in other cases it is a cryptic and not-helpful pop-up text such as “CGM2011”, “CGM PDF”, “COGEM”, “CGM 2011-07 Bijlage I Algemene text”, without a hyperlink. 
  7. Pseudomonas aeruginosa: the field Plant Pathogen has a link to a personal file ( file:///Users/catherineng/Downloads/54028.pdf) that does not work for other users. 
  8. Haloferax denitrificans: another personal link: file:///C:/Users/Maddie/Downloads/35960.pdf
  9. Human herpesvirus 4: None of the yellow I’s appear to show any text.
  10. Some of the listed sources of information consists of 2 URLs separated by a space; it is tricky to correctly copy/paste these into a browser. E.g. Methanobrevibacter smithii: “https://microbewiki.kenyon.edu/index.php/Methanobrevibacter_Smithii http://bacmap.wishartlab.com/organisms/525” 
  11. The i under Bacillus antracis lists about 10 URLs but most are private search terms, so they are not useful for anyone. The complete list is: “http://medschool.creighton.edu/fileadmin/user/medicine/MMI/Files/Bacteria_Table.pdf ; http://www.life.umd.edu/classroom/bsci424/PathogenDescriptions/PathogenList.htm#D; https://www.patricbrc.org/portal/portal/patric/SpecialtyGeneList?cType=genome&cId=1297866.3&kw=source:%22Victors%22 ; http://ardb.cbcb.umd.edu/cgi/search.cgi?db=R&term=YP_001373621 ; http://ardb.cbcb.umd.edu/cgi/search.cgi?db=R&term=ZP_02395450 ; http://ardb.cbcb.umd.edu/cgi/search.cgi?db=R&term=ZP_02391336 ; http://ardb.cbcb.umd.edu/cgi/search.cgi?db=R&term=ZP_03108029 ; https://microbewiki.kenyon.edu/index.php/Bacillus ; https://en.wikipedia.org/wiki/Bacillus_anthracis ; https://gold.jgi.doe.gov/organisms?id=3251”
5. Layout:
  1. I found the use of upper and lower case for the microorganism names a bit distracting. Bacteria are listed as completely lower case in the dark top bar, but in upper case in the bottom part. This might be great from a designer point of view, but it is not how most of us are used to write bacterial names. 
  2. When browsing taxonomically, viruses are listed in non-alphabetical order, making it hard to find the correct entry. E.g. Picornaviridae-Enterovirus or genus Bacillus both lead to such a list.
  3. In “Optimal Temerature” there is a “P” missing
  4. The name of the category called “Microbiome location “ suggests this field would contain a location, such as gut/mouth/skin), while it is a yes/no field. 
6.  Suggestion for a future additions:
  1. a short line describing what the organism is known for.
  2. Additional categories: chromosome information (linear, circular, how many, number of ribosomal operon copies, RNA/DNA for viruses), use in food industry (brewing, bread making, probiotic)

I have read this submission. I believe that I have an appropriate level of expertise to confirm that it is of an acceptable scientific standard, however I have significant reservations, as outlined above.

## References

[1] PaceNR: Time for a change. *Nature*.2006;441(7091) : 10.1038/441289a 289 10.1038/441289a 16710401

[2] StaabJFBradwaySDFidelPLSundstromP: Adhesive and mammalian transglutaminase substrate properties of Candida albicans Hwp1. *Science*.1999;283(5407) :1535-8 1006617610.1126/science.283.5407.1535

# Peer review: Referee response for version 1

**DOI:** 10.21956/gatesopenres.13832.r26309

**Published:** 2018-03-15

**Authors:** Nicole M. Vega

**Affiliations:** 1 Biology Department, Emory University, Atlanta, GA, USA

In this manuscript, the authors describe the creation and construction of the Microbe Directory, a resource for profiling and annotating species after large-scale metagenomic taxonomic analyses.

 I very much like the idea of the Microbe Directory and think that this could be a valuable resource for the field. The manuscript describing the Directory’s construction and curation to date was clear and understandable.

 I think that the ability of researchers to add information to the database directly is a great feature. Is there also a plan and/or schedule for incorporating database updates from the sources described in the paper?

 I did not download the database, but I did try the web interface and found it fairly intuitive to use. The Browse function was a little odd - it would be helpful if the options for each clade were presented alphabetically or in some other obvious order.

I have read this submission. I believe that I have an appropriate level of expertise to confirm that it is of an acceptable scientific standard.

# Peer review: Referee response for version 1

**DOI:** 10.21956/gatesopenres.13832.r26238

**Published:** 2018-03-15

**Authors:** James E. McDonald

**Affiliations:** 1 School of Biological Sciences, Bangor University, Bangor, UK

**Concept:**

 The microbe directory is an excellent concept and aims to provide phenotypic and ecological profiles of approx. 7500 microbial species represented in the MetaPhlAn2 database. Although some information is present in other repositories, the Microbe Directory aggregates information on the functional, biological or medical traits of these organisms into a single source where the profiles may be further expanded to represent a useful resource to better interpret the functional and ecological properties of taxonomic data. If the directory continues to grow and expand with additional information, it would be a fantastic and heavily-utilised resource for the wider community. In particular, integration of data for bacteria, archaea, viruses, fungi, and protozoa in the same database is a positive strategy.

 An important feature of the Microbe Directory is that while there is always the potential for human error in manually curated databases, scientists can generate an account and submit edits and changes to the information hosted in the database. I hope that the wider scientific community engages with and contributes to the directory in order to enable it to reach its potential as an important resource for microbiologists.

**Manuscript:**

 Abstract. The abstract focusses heavily on the application of the directory ‘downstream of large-scale metagenomic taxonomic analysis’ and ‘designed to serve as a resource for researchers conducting metagenomic analysis’, but perhaps this is too narrow a focus on the utility of the directory. I can see several other uses for the directory in other areas of microbiology; to inform/validate the potential phenotypic and ecological properties of a microbial isolate, or as an information source on a specific microorganism for an undergraduate student after a lab class, for example. Maybe it’s worth re-wording this to broaden the potential for wider adoption of the resource.

 Average values. Table 1 describes the microbial features currently listed in the directory. However, in instances where more than one optimal temperature and pH could be found for different strains of a species, an average value has been taken. This would mask the range of optimal temperatures across the strains, which is useful information if you are using the resource to find out the best temperature(s) to grow a species at. Could the range of temperature recorded not also be provided as an additional source of information?  

**Website and sources of information:**

 The website looks good and was generally easy to navigate.

 Information. I performed a few searches for microorganisms that we work on, some of which are not well-characterised at present, and was not surprised to see that for most of these many of the categories were blank. However, I then looked at some very well-studied organisms and also found lots of blank categories. For example, only 3/11 categories were complete for Bacillus subtilis, when a quick google search reveals primary research articles that provide information on several of these categories (e.g. that it is a spore former). E. coli also has no data for biofilm formation, which can again be verified with a quick google search. Going forward, additional buy-in will therefore been needed to ensure that the information is complete as possible.

 For some species, the information links didn’t work (I got an ‘error 404’ code) which made it difficult to find the source of information, but others worked fine.

 Where the links did work, many of the sources of information were webpages (e.g. wiki pages) that did refer to primary literature that could be accessed to verify the information. However, in my view, if it was possible to provide links to more than one reference in the primary literature, and to allow others to add links to primary research articles, you could very quickly generate a set of primary literature that described those key attributes, which would be very beneficial as a reference source and for validation of the information.

 Some links to information about microbial species took me to website articles that although were apparently peer-reviewed, had collated information from primary research articles. However, if it is possible to incorporate them, direct links to the research articles themselves would in my view be more useful.

I have read this submission. I believe that I have an appropriate level of expertise to confirm that it is of an acceptable scientific standard, however I have significant reservations, as outlined above.

# Peer review: Referee response for version 1

**DOI:** 10.21956/gatesopenres.13832.r26191

**Published:** 2018-01-12

**Authors:** David A. Coil

**Affiliations:** 1 Genome Center, University of California, Davis, Davis, CA, USA

I love the idea behind “The Microbe Directory”. I think this information will be of great value and I really like the way it was generated with the help of students. With the ability to expand the database, I think this could become an important resource. In particular I’m curious if the authors have considered working with the folks at Traitar? They have a really useful tool but their underlying phenotypic data is lacking and is heavily biased towards human pathogens in a way that the Microbe Directory appears not to be.

 However, my attempts to use the website were somewhat frustrating. It’s not clear to me in a review of this nature whether I should be reviewing just the paper (basically fine) or the software itself (needs work before going live).

**Paper**

 The paper is well-written and clear. I only had very minor suggestions (harder without line numbers!)

 The phrase starting with “these features were considered” in the “Building the microbe directory section needs grammatical revision.

 In the same section the sentence “These peer-reviewed resources and databases…” is a bit misleading since most of the listed resources are not peer-reviewed.

 Not sure the “(RegEx)” abbreviation is useful since it’s never used again.

 “The edits are then put in a queue to be later reviewed by the Microbe Directory team”. I would like to see a bit more about what the criteria for review are. Will they just be checking for spam or will they actually verify information using the reference(s)?

**Website**

 The site is very clean and easy to navigate. However, when I attempted to do things I encountered some snags.

 Firstly, I went and looked at some microbes. The first think I noticed is that reference links aren’t clickable links, they are just html which is a bit off-putting and requires careful copying and pasting. But in a case where there are multiple links it becomes a mess (see screenshot). It seems like some better way to parse links is required, and having them be clickable would be awesome. The first one I tried also led to a 404 error, is there some way that links could be automatically checked?

 So after I created an account I clicked on “Contribute” which took me to the login page, after logging in, it dropped me back at the main page which seems not ideal… I then had to navigate back to the microbe I was interested in. Then after clicking on “Contribute”, I’m faced with some fields… the first of which is the Microbe ID… but it doesn’t say that anywhere, there’s just a number there. A bit confusing. Perhaps this field could be labeled?

 Then I wanted to add something about Gram staining… but there’s no key for the “Values” field (see screenshot). I had to open a new window and pull up another organisms to know that “1” is what I wanted for Gram-positive. Is it possible to display the key for the values on the contribute page? Or do have a small key appear for the selected attribute? Some way for someone to have access to the key within the “Contribute” page.

 I was surprised to see an entry like “ *Porphyrobacter* sp AAP82”. Is there a rationale for including isolates that don’t have a species level identification? Not sure how useful this sort of thing is for the stated purpose of the database. For example in this case there are only two pieces of data, the COGEM listing (which since the reference “link” just says “COGEM” can’t be verified) and the Gram stain field which links to the listing for that genus at bacterio.net which actually doesn’t contain information about Gram staining and anyway wouldn’t have a listing for an isolate like this that doesn’t have a species name. How does something like this end up in the database? 

 In a similar vein, there are microbes in the database that have no information whatsoever, is this expected?

 I really wanted to try adding a new species to the database, but couldn’t find any way to do so. The paper sort of implies that this is possible but I didn’t see any such option on the website?  I then went to the “Contact” page figuring that I would send a ping with this question. But there’s no general contact address for the project? Seems like that might be useful? Wasn’t sure which specific person would be appropriate for this question.

I have read this submission. I believe that I have an appropriate level of expertise to confirm that it is of an acceptable scientific standard, however I have significant reservations, as outlined above.

# Response

**Published:** 2018-02-04

**Authors:** Heba Shaaban

**Affiliations:** Weill Cornell Medical Center, USA

Thank you for your review, Dr. Coil.

Paper:

The edits you suggested regarding the paper will be published on version 2 of the manuscript. 

As for the edits that contributors will be making to the site, we will review them one-by-one to confirm that the sources/citations submitted are reliable and that the data inputted is verified by the reference. We have an administrative view that our team uses to accept/reject edits. We will expand on this in the paper, as suggested.

Website:

We are sorry that you had a difficult experience using our website. The links are now clickable if they are validated, and you should be redirected to the link right away. If more than one link was cited, you can click on the icon to see all the links. For now, copying and pasting the links (if multiple) is the only way to view them. We eventually want to change this, but due to the non-standard format of some of the citations, this is not currently possible. We will need to manually re-curate the citations in order to provide this functionality, and we are planning to do so in the near future. As for the link you clicked on being expired, the project has been ongoing for about two years now and some of the links are indeed broken. We plan to add timestamps to each citation, but this is currently not implemented. If a link is broken, it is possible for Microbe Directory users to manually submit edits using the “Contribute” interface.

We also programmed the site to redirect you to the microbe you were attempting to edit after you create an account. Thank you for bringing that to our attention. The microbe ID was actually an in-house cataloging number for admins and we understand why it might be confusing. It is just meant to make it easier to share species on social media without having to type in the species name. Also, the key for values will now appear on the contribute page. We also created a drop-down menu for each value, which should make it easier to edit.

As for including isolates that don’t have a species-level identification, we made the decision to be compatible with MetaPhlAn2, so researchers using this popular metagenomics analysis tool would have their code work “out-of-the-box.” If in the future, researchers decide to work with these strains or species, they will already be cataloged in the database. We are aware that there are microbes for which there is no information. We wanted to include these to be conformant to MetaPhlAn2 and also to provide a scaffold for users to fill in additional information.

Additionally, users can now add new species to the database by using the link on the “Contribute” page. We also updated the contact page to include our role descriptions in the project, so that we may be contacted accordingly. We also improved our "Help" page to include more instructions on contributing and to address some troubleshooting. 

Lastly, this is just version 1 of the Microbe Directory. As the project expands, we plan to make changes to the site format and configuration as necessary in addition to maintaining proper quality-control. As you had mentioned with Traitar, we want to collaborate with organizations that have their own databases and incorporate them into the Microbe Directory. We think v1 of the Microbe Directory provides a scaffold for expansion, and we really want to see this project grow over time.

Thank you for your time and efforts in reviewing our project.
